# Supplementary material for: Leaf Functional Traits and Their Influencing Factors in Six Typical Vegetation Communities
Source: Plants (Basel). 2024 Aug 30;13(17):2423. doi: 10.3390/plants13172423 (PMC11397209; doi:10.3390/plants13172423)
Supplement: Supplementary file 1 [file plants-13-02423-s001.zip › Table S1.pdf]

Table S1: The Species Checklist of the Chayu River Basin

No.	species	genus	family	order
1	*Hymenidium chloroleucum* (Diels) Pimenov & Kljuykov	Hymenophyllum	Hymenophyllaceae	hymenophyllales
2	*Pteris dactylina* Hook.	Pteris	Pteridaceae	polypodiales
3	*Coniogramme japonica* (Thunb.) Diels	Coniogramme	Pteridaceae	polypodiales
4	*Pteridium aquilinum var. latiusculum* (Desv.) Underw. ex A. Heller	Pteridium	Dennstaedtiaceae	polypodiales
5	*Micranthes melanocentra* (Franch.) Losinsk.	Microlepia	Dennstaedtiaceae	polypodiales
6	*Asplenium pseudolaserpitiifolium* Ching	Asplenium	Aspleniaceae	polypodiales
7	*Pronephrium simplex* (Hook.) Holttum	Pronephrium	Thelypteridaceae	polypodiales
8	*Athyrium subtriangulare* Bedd.	Athyrium	Athyriaceae	polypodiales
9	*Pyrrosia calvata* (Baker) Ching	Pyrrosia	Polypodiaceae	polypodiales
10	*Polygonatum verticillatum* (L.) All.	Polypodiodes	Polypodiaceae	polypodiales
11	*Polystichum otophorum* (Franch.) Bedd.	Polystichum	Dryopteridaceae	polypodiales
12	*Dryopteris barbigera* (T. Moore & Hook.) Kuntze	Dryopteris	Dryopteridaceae	polypodiales
13	*Abies chayuensis* W. C. Cheng & L. K. Fu	Abies	Pinaceae	pinales
14	*Pinellia ternate* (Thunb.) Ten. ex Breitenb.	Pinus	Pinaceae	pinales

15	*Juncus thomsonii* Buchenau	Juniperus	Cupressaceae	cupressales
16	*Juniperus convallium* Rehder & E. H. Wilson	Juniperus	Cupressaceae	cupressales
17	*Pedicularis muscicola* Maxim.	Peperomia	Piperaceae	piperales
18	*Ligustrum sinense* Lour.	Litsea	Lauraceae	lauracles
19	*Cinnamomum cassia* (L.) D. Don	Cinnamomum	Lauraceae	lauracles
20	*Picris hieracioides* L.	Pinellia	Araceae	alismatales
21	*Arisaema elephas* Buchet	Arisaema	Araceae	alismatales
22	*Dioscorea bulbifera* L.	Dioscorea	Dioscoreaceae	dioscoreales
23	*Smilax menispermoidea* A. DC.	Smilax	Smilacaceae	liliales
24	*Fritillaria delavayi* Franch.	Fritillaria	Liliaceae	liliales
25	*Streptopus simplex* D. Don	Streptopus	Liliaceae	liliales
26	*Microlepia strigose* (Thunb.) C. Presl	Neottianthe	Orchidaceae	asparagales
27	*Goniophlebium amoenum* (Wall. ex Mett.) Bedd.	Habenaria	Orchidaceae	asparagales
28	*Epipactis helleborine* (L.) Crantz	Epipactis	Orchidaceae	asparagales
29	*Maianthemum purpureum* (Wall.) LaFrankie	Malaxis	Orchidaceae	asparagales
30	*Imperata cylindrica* (L.) P. Beauv.	Iris	Iridaceae	asparagales
31	*Allium kingdonii* Stearn	Allium	Amaryllidaceae	asparagales
32	*Allium sikkimense* Baker	Allium	Amaryllidaceae	asparagales
33	*Odontostemma fridericae* (Hand.-	Ophiopogon	Asparagaceae	asparagales

	Mazz.) Sadeghian & Zarre			
34	*Lyonia villosa* (Wall. ex C. B. Clarke) Hand.-Mazz.	Maianthemum	Asparagaceae	asparagales
35	*Poa polycolea* Stapf in Hook f.	Polygonatum	Asparagaceae	asparagales
36	*Polygonatum oppositifolium* (Wall.) Royle	Polygonatum	Asparagaceae	asparagales
37	*Juglans sigillata* Dode	Juncus	Juncaceae	poales
38	*Carex laeta*B oott	Carex	Cyperaceae	poales
39	*Carex kokanica* (Regel) S. R. Zhang	Carex	Cyperaceae	poales
40	*Carex leiorhyncha* C. A. Mey.	Carex	Cyperaceae	poales
41	*Carex kansuensis* Nelmes	Carex	Cyperaceae	poales
42	*Piptatherum munroi* (Stapf) Mez	Poa	Poaceae	poales
43	*Iris goniocarpa* Baker	Isachne	Poaceae	poales
44	*Impatiens fragicolor* C. Marquand & Airy Shaw	Imperata	Poaceae	poales
45	*Ptilagrostis mongholica* (Turcz. ex Trin.) Griseb. in Ledeb.	Ptilagrostis	Poaceae	poales
46	*Piptanthus nepalensis* auct. non (Hook.) D. Don: Turner	Piptatherum	Poaceae	poales
47	*Piptatherum laterale* (Regel) Munro ex Nevski	Piptatherum	Poaceae	poales
48	*Elymus dolichatherus* (Keng) S. L. Chen	Elymus	Poaceae	poales
49	*Festuca ovina* L.	Festuca	Poaceae	poales

50	*Trisetum clarkei* (Hook. f.) R. R. Stewart	Trisetum	Poaceae	poales
51	*Calamagrostis pseudophragmites* (Haller f.) Koeler	Calamagrostis	Poaceae	poales
52	*Mallotus tenuifolius* Pax	Meconopsis	Papaveraceae	ranunculales
53	*Corydalis calcicole* W. W. Sm.	Corydalis	Papaveraceae	ranunculales
54	*Corydalis melanochlora* Maxim.	Corydalis	Papaveraceae	ranunculales
55	*Corydalis benecincta* W. W. Sm.	Corydalis	Papaveraceae	ranunculales
56	*Circaeaster agrestis* Maxim.	Circaeaster	Circaeasteraceae	ranunculales
57	*Sinopodophyllum hexandrum* (Royle) T. S. Ying	Sinopodophyll um	Berberidaceae	ranunculales
58	*Berberis franchetiana* C. K. Schneid.	Berberis	Berberidaceae	ranunculales
59	*Berberis pruinose* Franch.	Berberis	Berberidaceae	ranunculales
60	*Berberis tsarongensis* Stapf	Berberis	Berberidaceae	ranunculales
61	*Berberis diaphana* Maxim.	Berberis	Berberidaceae	ranunculales
62	*Thalictrum delavayi* Franch.	Thalictrum	Ranunculaceae	ranunculales
63	*Thalictrum alpinum* L.	Thalictrum	Ranunculaceae	ranunculales
64	*Delphinium tangkulaense* W. T. Wang	Delphinium	Ranunculaceae	ranunculales
65	*Delphinium brunonianum* Royle	Delphinium	Ranunculaceae	ranunculales
66	*Trollius yunnanensis* (Franch.) Ulbr.	Trollius	Ranunculaceae	ranunculales
67	*Trollius farreri* Stapf	Trollius	Ranunculaceae	ranunculales
68	*Anemone rivularis* Buch.-Ham. ex DC.	Anemone	Ranunculaceae	ranunculales

69	*Clematis heracleifolia* DC.	Clematis	Ranunculaceae	ranunculales
70	*Ranunculus tanguticus* (Maxim.) Ovcz.	Ranunculus	Ranunculaceae	ranunculales
71	*Ranunculus glareosus* Hand.-Mazz.	Ranunculus	Ranunculaceae	ranunculales
72	*Buxus Myrica* H. Lév.	Buxus	Buxaceae	buxales
73	*Sinocrassula indica* (Decne.) A. Berger	Sinocrassula	Crassulaceae	saxifragales
74	*Rhodiola bupleuroides* (Wall. ex Hook. f. & Thomson) S. H. Fu	Rhodiola	Crassulaceae	saxifragales
75	*Rhodiola fastigiate* (Hook. f. & Thomson) S. H. Fu	Rhodiola	Crassulaceae	saxifragales
76	*Ribes orientale* Desf.	Ribes	Grossulariaceae	saxifragales
77	*Ribes glaciale* Wall.	Ribes	Grossulariaceae	saxifragales
78	*Meconopsis racemosa* Maxim.	Micranthes	Saxifragaceae	saxifragales
79	*Saxifraga finitima* W. W. Sm.	Saxifraga	Saxifragaceae	saxifragales
80	*Geranium pylzowianum* Maxim.	Geranium	Geraniaceae	geraniales
81	*Geranium himalayense* Klotzsch	Geranium	Geraniaceae	geraniales
82	*Circaea alpina* L.	Circaea	Onagraceae	myrtales
83	*Epilobium sikkimense* Hausskn.	Epilobium	Onagraceae	myrtales
84	*Rhus chinensis* Mill.	Rhus	Anacardiaceae	sapindales
85	*Acer stachyophyllum subsp. Betulifolium* (Maxim.) P. C. DeJong	Acer	Sapindaceae	sapindales
86	*Daphne tangutica* Maxim.	Daphne	Thymelaeaceae	malvales
87	*Cardamine griffithii* Hook. f. & Thomson	Cardamine	Brassicaceae	brassicales

88	*Cardamine occulta* Hornem.	Cardamine	Brassicaceae	brassicales
89	*Draba eriopoda* Turcz. ex Ledeb.	Draba	Brassicaceae	brassicales
90	*Eutrema verticillatum* (Jeffrey & W. W. Sm.) Al-Shehbaz & Warwick	Eutrema	Brassicaceae	brassicales
91	*Eutrema deltoideum* (Hook. f. & Thomson) O. E. Schulz	Eutrema	Brassicaceae	brassicales
92	*Parnassia delavayi* Franch.	Parnassia	Parnassiaceae	celastrales
93	*Parasenecio quinquelobus* (Wall. ex DC.) Y. L. Chen	Parnassia	Parnassiaceae	celastrales
94	*Malaxis monophyllos* (L.) Sw.	Mallotus	Euphorbiaceae	malpighiales
95	*Vernicia fordii* (Hemsl.) Airy Shaw	Vernicia	Euphorbiaceae	malpighiales
96	*Euphorbia stracheyi* Boiss.	Euphorbia	Euphorbiaceae	malpighiales
97	*Viola szetschwanensis* W. Becker & H. Boissieu	Viola	Violaceae	malpighiales
98	*Viola biflora var. rockiana* (W. Becker) Y. S. Chen	Viola	Violaceae	malpighiales
99	*Populus davidiana* Dode	Populus	Salicaceae	malpighiales
100	*Salix rehderiana* C. K. Schneid. in Sargent	Salix	Salicaceae	malpighiales
101	*Pinus yunnanensis* Franch.	Piptanthus	Fabaceae	fabales
102	*Apios carnea* (Wall.) Benth. ex Baker	Apios	Fabaceae	fabales
103	*Apios delavayi* Franch.	Apios	Fabaceae	fabales

104	*Tibetia himalaica* (Baker) H. P. Tsui	Tibetia	Fabaceae	fabales
105	*Hedera nepalensis var. sinensis* (Tobler) Rehder	Hedysarum	Fabaceae	fabales
106	*Ophiopogon bodinieri* H. Lév.	Oxytropis	Fabaceae	fabales
107	*Quercus spinosa* David ex Franch.	Quercus	Fagaceae	fagales
108	*Quercus aquifolioides* Rehder & E. H. Wilson in Sarg.	Quercus	Fagaceae	fagales
109	*Quercus oxyodon* Miq.	Quercus	Fagaceae	fagales
110	*Isachne globosa* (Thunb.) Kuntze	Juglans	Juglandaceae	fagales
111	*Betula utilis* D. Don	Betula	Betulaceae	fagales
112	*Betula platyphylla* Sukaczev	Betula	Betulaceae	fagales
113	*Celtis koraiensis* Nakai	Celtis	Cannabaceae	rosales
114	*Ficus tikoua* Bureau	Ficus	Moraceae	rosales
115	*Prunus conadenia* Koehne in Sarg.	Prunus	Rosaceae	rosales
116	*Spiraea alpina* Pall.	Spiraea	Rosaceae	rosales
117	*Spiraea arcuate* Hook. f.	Spiraea	Rosaceae	rosales
118	*Sorbus rehderiana* Koehne in Sarg.	Sorbus	Rosaceae	rosales
119	*Cotoneaster rubens* W. W. Sm.	Cotoneaster	Rosaceae	rosales
120	*Cotoneaster rotundifolius* Wall. ex Lindl.	Cotoneaster	Rosaceae	rosales
121	*Cotoneaster microphyllus* Wall. ex Lindl.	Cotoneaster	Rosaceae	rosales
122	*Rubus stans* Focke	Rubus	Rosaceae	rosales
123	*Rosa omeiensis* Rolfe	Rosa	Rosaceae	rosales
124	*Rosa omeiensis f. pteracantha* Rehd.et Wils.	Rosa	Rosaceae	rosales

125	*Rosa omeiensis f. paucijuga* Yü et Ku	Rosa	Rosaceae	rosales
126	*Agrimonia pilosa var. nepalensis* (D. Don) Nakai	Agrimonia	Rosaceae	rosales
127	*Argentina tugitakensis* (Masam.) Soják	Argentina	Rosaceae	rosales
128	*Cotoneaster multiflorus* Bunge in Ledeb.	Cotoneaster	Rosaceae	rosales
129	*Potentilla saundersiana* Royle	Potentilla	Rosaceae	rosales
130	*Dasiphora glabra* (G. Lodd.) Soják	Dasiphora	Rosaceae	rosales
131	*Fragaria nubicola* (Hook. f.) Lindl. ex Lacaita	Fragaria	Rosaceae	rosales
132	*Sibbaldia cuneata* Hornem. ex Kuntze	Sibbaldia	Rosaceae	rosales
133	*Sibbaldia purpurea* Royle	Sibbaldia	Rosaceae	rosales
134	*Achyranthes aspera* L.	Achyranthes	Amaranthaceae	caryophyllales
135	*Silene nigrescens* (Edgew.) Majumdar	Silene	Caryophyllaceae	caryophyllales
136	*Odontostemma barbatum* (Franch.) Sadeghian & Zarre	Odontostemma	Caryophyllaceae	caryophyllales
137	*Neottianthe cucullate* (L.) Schltr.	Odontostemma	Caryophyllaceae	caryophyllales
138	*Rheum pumilum* Maxim.	Rheum	Polygonaceae	caryophyllales
139	*Rheum australe* D. Don	Rheum	Polygonaceae	caryophyllales
140	*Rheum spiciforme* Royle	Rheum	Polygonaceae	caryophyllales
141	*Peperomia tetraphylla* (G. Forst.) Hook. & Arn.	Persicaria	Polygonaceae	caryophyllales
142	*Juniperus tibetica* Kom.	Koenigia	Polygonaceae	caryophyllales

143	*Koenigia hookeri* (Meisn.) T. M. Schust. & Reveal	Koenigia	Polygonaceae	caryophyllales
144	*Bistorta vivipara* (L.) Gray	Bistorta	Polygonaceae	caryophyllales
145	*Bistorta macrophylla* (D. Don) Soják	Bistorta	Polygonaceae	caryophyllales
146	*Bistorta suffulta subsp. Pergracilis* (Hemsl.) Soják	Bistorta	Polygonaceae	caryophyllales
147	*Dichroa febrifuga* Lour.	Dichroa	Hydrangeaceae	cornales
148	*Heracleum candicans* Wall. ex DC.	Hydrangea	Hydrangeaceae	cornales
149	*Petasites tricholobus* Franch.	Philadelphus	Hydrangeaceae	cornales
150	*Deutzia hookeriana* (C. K. Schneid.) Airy Shaw	Deutzia	Hydrangeaceae	cornales
151	*Impatiens arguta* Hook. f. & Thomson	Impatiens	Balsaminaceae	ericales
152	*Ilex dipyrena* Wall.	Impatiens	Balsaminaceae	ericales
153	*Diospyros kaki var. silvestris* Makino	Diospyros	Ebenaceae	ericales
154	*Embelia vestita* Roxb.	Embelia	Primulaceae	ericales
155	*Primula alpicola* (W. W. Sm.) Stapf	Primula	Primulaceae	ericales
156	*Symplocos lancifolia* Siebold & Zucc.	Symplocos	Symplocaceae	ericales
157	*Saurauia tristyla* DC.	Saurauia	Actinidiaceae	ericales
158	*Pyrola atropurpurea* Franch.	Pyrola	Ericaceae	ericales
159	*Lonicera tangutica* Maxim.	Lyonia	Ericaceae	ericales
160	*Vaccinium bulleyanum* (Diels) Sleum.	Vaccinium	Ericaceae	ericales
161	*Rhododendron wardii* W. W. Sm.	Rhododendron	Ericaceae	ericales

162	*Rhododendron oreotrephes* W. W. Sm.	Rhododendron	Ericaceae	ericales
163	*Rhododendron triflorum* Hook.	Rhododendron	Ericaceae	ericales
164	*Rhododendron rubiginosum* Franch.	Rhododendron	Ericaceae	ericales
165	*Damnacanthus indicus* C. F. Gaertn.	Damnacanthus	Rubiaceae	gentianales
166	*Leontopodium souliei* Beauverd	Leptodermis	Rubiaceae	gentianales
167	*Galium bungei* Steud.	Galium	Rubiaceae	gentianales
168	*Habenaria aitchisonii* Rchb. f.	Halenia	Gentianaceae	gentianales
169	*Swertia zayuensis* T. N. Ho & S. W. Liu	Swertia	Gentianaceae	gentianales
170	*Gentiana otophora* Franch.	Gentiana	Gentianaceae	gentianales
171	*Gentiana bella* Franch.	Gentiana	Gentianaceae	gentianales
172	*Gentiana crassuloides* Bureau & Franch.	Gentiana	Gentianaceae	gentianales
173	*Gentiana algida* Pall.	Gentiana	Gentianaceae	gentianales
174	*Ehretia acuminata* R. Br.	Ehretia	Boraginaceae	boraginales
175	*Chionocharis hookeri* (C. B. Clarke) I. M. Johnst.	Chionocharis	Boraginaceae	boraginales
176	*Trigonotis tibetica* (C. B. Clarke) I. M. Johnst.	Trigonotis	Boraginaceae	boraginales
177	*Ligularia tsangchanensis* (Franch.) Hand.-Mazz.	Ligustrum	Oleaceae	lamiales
178	*Koenigia nummulariifolia* (Meisn.) Měsíček & Soják	Lagotis	Plantaginaceae	lamiales
179	*Veronica szechuanica* Batalin	Veronica	Plantaginaceae	lamiales

180	*Strobilanthes kingdonii* J. R. I. Wood	Strobilanthes	Acanthaceae	lamiales
181	*Eriophyton wallichii* Benth.	Eriophyton	Lamiaceae	lamiales
182	*Elsholtzia fruticose* (D. Don) Rehder	Elsholtzia	Lamiaceae	lamiales
183	*Salvia wardii* E. Peter	Salvia	Lamiaceae	lamiales
184	*Salvia 'Waverly'*	Salvia	Lamiaceae	lamiales
185	*Pedicularis confertiflora* Prain	Pedicularis	Orobanchaceae	lamiales
186	*Pedicularis cryptantha* C. Marquand & Airy Shaw	Pedicularis	Orobanchaceae	lamiales
187	*Pedicularis likiangensis* Franch. ex Maxim.	Pedicularis	Orobanchaceae	lamiales
188	*Pedicularis mollis* Wall.	Pedicularis	Orobanchaceae	lamiales
189	*Pedicularis elwesii* Hook. f.	Pedicularis	Orobanchaceae	lamiales
190	*Parnassia yunnanensis* Franch.	Pedicularis	Orobanchaceae	lamiales
191	*Hymenophyllum badium* Hook. & Grev.	Ilex	Aquifoliaceae	aquifoliales
192	*Sanicula chinensis* Bunge	Sanicula	Apiaceae	apiales
193	*Chamaesium paradoxum* H. Wolff	Chamaesium	Apiaceae	apiales
194	*Hydrangea heteromalla* D. Don	Hymenidium	Apiaceae	apiales
195	*Heptapleurum arboricola* Hayata	Heracleum	Apiaceae	apiales
196	*Hedysarum sikkimense* auct. non Benth. ex Baker: P. C. Li	Heptapleurum	Araliaceae	apiales
197	*Brassaiopsis palmata* (Roxb.) Kurz	Brassaiopsis	Araliaceae	apiales

198	*Brassaiopsis triloba* K. M. Feng	Brassaiopsis	Araliaceae	apiales
199	*Halenia elliptica* D. Don	Hedera	Araliaceae	apiales
200	*Eleutherococcus wilsonii* (Harms) Nakai	Eleutherococcus	Araliaceae	apiales
201	*Adoxa moschatellina* L.	Adoxa	Viburnaceae	dipsacales
202	*Viburnum grandiflorum* Wall. ex DC.	Viburnum	Viburnaceae	dipsacales
203	*Viburnum atrocyaneum* C. B. Clarke	Viburnum	Viburnaceae	dipsacales
204	*Acanthocalyx nepalensis* (D. Don) M. J. Cannon	Acanthocalyx	Caprifoliaceae	dipsacales
205	*Triplostegia glandulifer* Wall. ex DC.	Triplostegia	Caprifoliaceae	dipsacales
206	*Litsea pungens* Hemsl.	Lonicera	Caprifoliaceae	dipsacales
207	*Lonicera rupicola* Hook. f. & Thomson	Lonicera	Caprifoliaceae	dipsacales
208	*Lonicera angustifolia var. myrtillus* (Hook. f. & Thomson) Q. E. Yang	Lonicera	Caprifoliaceae	dipsacales
209	*Lonicera scabrida* Franch.	Lonicera	Caprifoliaceae	dipsacales
210	*Lonicera nigra* L.	Lonicera	Caprifoliaceae	dipsacales
211	*Lonicera hispida* Pall. ex Roem. & Schult.	Lonicera	Caprifoliaceae	dipsacales
212	*Cyananthus hookeri* C. B. Clarke	Cyananthus	Campanulaceae	asterales
213	*Codonopsis foetens* Hook. f. & Thomson	Codonopsis	Campanulaceae	asterales
214	*Pseudocodon convolvulaceus subsp. Forrestii* (Diels) D. Y. Hong	Pseudocodon	Campanulaceae	asterales

215	*Lagotis integra* W. W. Sm.	Leibnitzia	Asteraceae	asterales
216	*Saussurea picridifolia* (Hand.-Mazz.) Y. S. Chen & Qian Yuan	Saussurea	Asteraceae	asterales
217	*Saussurea tridactyla* Sch. Bip. ex Hook. f.	Saussurea	Asteraceae	asterales
218	*Saussurea salwinensis* Anthony	Saussurea	Asteraceae	asterales
219	*Saussurea hieracioides* Hook. f.	Saussurea	Asteraceae	asterales
220	*Saussurea pachyneura* Franch.	Saussurea	Asteraceae	asterales
221	*Saussurea przewalskii* Maxim.	Saussurea	Asteraceae	asterales
222	*Ainsliaea latifolia* (D. Don) Sch. Bip.	Ainsliaea	Asteraceae	asterales
223	*Philadelphus tomentosus* Wall. ex G. Don	Picris	Asteraceae	asterales
224	*Youngia racemifera* (Hook. f.) Babc. & Stebbins	Youngia	Asteraceae	asterales
225	*Bidens Pilosa* L.	Bidens	Asteraceae	asterales
226	*Galinsoga parviflora* Cav.	Galinsoga	Asteraceae	asterales
227	*Leibnitzia nepalensis* (Kunze) Kitam.	Leontopodium	Asteraceae	asterales
228	*Leontopodium nanum* (Hook. f. & Thomson ex C. B. Clarke) Hand.-Mazz.	Leontopodium	Asteraceae	asterales
229	*Anaphalis flavescens* Hand.-Mazz.	Anaphalis	Asteraceae	asterales
230	*Anaphalis margaritacea var. angustifolia* (Franch. & Sav.) Hayata	Anaphalis	Asteraceae	asterales

231	*Erigeron patentisquama* Jeffrey	Erigeron	Asteraceae	asterales
232	*Aster diplostephioides* (DC.) Benth. ex C. B. Clarke	Aster	Asteraceae	asterales
233	*Aster himalaicus* C. B. Clarke	Aster	Asteraceae	asterales
234	*Synotis erythropappa* (Bureau & Franch.) C. Jeffrey & Y. L. Chen	Synotis	Asteraceae	asterales
235	*Persicaria jucunda* (Meisn.) Migo	Petasites	Asteraceae	asterales
236	*Leptodermis potaninii var. glauca* (Diels) H. J. P. Winkler	Ligularia	Asteraceae	asterales
237	*Oxytropis melanocalyx* Bunge	Parasenecio	Asteraceae	asterales
238	*Cremanthodium humile* Maxim.	Cremanthodium	Asteraceae	asterales
239	*Cremanthodium campanulatum* (Franch.) Diels	Cremanthodium	Asteraceae	asterales
240	*Cremanthodium decaisnei* C. B. Clarke	Cremanthodium	Asteraceae	asterales
241	*Cremanthodium ellisii* (Hook. f.) Kitam.	Cremanthodium	Asteraceae	asterales
