# Supplementary material for: Leaf Functional Traits and Their Influencing Factors in Six Typical Vegetation Communities
Source: Plants (Basel). 2024 Aug 30;13(17):2423. doi: 10.3390/plants13172423 (PMC11397209; doi:10.3390/plants13172423)
Supplement: Supplementary file 1 [file plants-13-02423-s001.zip › Table S2.pdf]

Table S2: Sampling Site Conditions in the Chayu River Basin

Community types	Sampling Site	Elevation/m	Longitude	Latitude
I	site1	1425	97.02747	28.36142
I	site2	1499	97.02592	28.36678
I	site3	1536	97.01184	28.36773
II	site4	2947	97.35453	29.04248
II	site5	2945	97.35058	29.04671
II	site6	2915	97.35299	29.04597
III	site7	3622	97.46972	28.69278
III	site8	3669	97.59139	28.79722
III	site9	3723	97.59028	28.79639
IV	site10	4309	97.74214	28.80857
IV	site11	4161	97.72972	28.81194
IV	site12	4204	97.73056	28.81278
V	site13	4552	97.70315	28.72834
V	site14	4583	97.70368	28.72799
V	site15	4566	97.70278	28.72889
VI	site16	4783	97.70964	28.72574
VI	site17	4756	97.70938	28.72466
VI	site18	4829	97.70793	28.72377

Community types: scle-rophyllous evergreen broad-leaved forests (I), temperate evergreen conif-erous forests (II), cold-temperate evergreen coniferous forests (III), alpine deciduous broad-leaved shrubs (IV), alpine meadows (V), and alpine scree sparse vegetation (VI).
