# Supplementary material for: Leaf Functional Traits and Their Influencing Factors in Six Typical Vegetation Communities
Source: Plants (Basel). 2024 Aug 30;13(17):2423. doi: 10.3390/plants13172423 (PMC11397209; doi:10.3390/plants13172423)
Supplement: Supplementary file 1 [file plants-13-02423-s001.zip › Table S3.pdf]

Table S3: Results of the Fourth-Corner Analysis

	LA	SLA	LT	LDMC	LNC	LCC	LPC	LKC
bio1	0.014*	0.320467	0.282074	0.336911	-0.015448276*	0.925662	0.237827	0.660545
bio2	0.150621	0.898154	0.014*	-0.02635294*	0.014*	0.319443	0.763259	0.362667
bio3	0.031111111*	0.102076	0.074667	0.113965	0.608	0.099556	0.07814	0.237827
bio4	-0.015448276*	0.320691	0.716066	0.674489	0.099556	0.918085	0.081455	0.970667
bio5	0.014*	0.336	0.258462	0.31915	-0.015448276*	0.957091	0.237827	0.634896
bio6	0.014*	0.349315	0.2624	0.320264	-0.015448276*	0.985802	0.237827	0.65664
bio7	-0.015448276*	0.421818	0.334164	0.362667	0.021*	0.931097	0.237827	0.876332
bio8	0.014*	0.316235	0.258462	0.320264	-0.015448276*	0.925662	0.247718	0.65664
bio9	0.014*	0.30975	0.320264	0.377354	-0.015448276*	0.914133	0.237827	0.720172
bio10	0.014*	0.316235	0.258462	0.320264	-0.015448276*	0.925662	0.247718	0.65664
bio11	0.014*	0.316235	0.319443	0.371597	-0.015448276*	0.918085	0.237827	0.716066
bio12	0.015448276*	0.224	0.592	0.65664	0.237827	0.449647	0.512883	0.839446
bio13	0.014*	0.216774	0.598297	0.661876	0.250047	0.425771	0.526247	0.839446
bio14	0.026352941*	0.163254	0.823258	0.839446	0.494734	0.258462	0.654667	0.570453
bio15	0.014*	0.240593	0.320691	0.512676	0.051692	0.654667	0.371597	0.813167
bio16	0.014*	0.217108	0.588549	0.65664	0.237827	0.442985	0.512883	0.839446
bio17	0.056	0.186667	0.918085	0.91363	0.7168	0.247718	0.880151	0.289761
bio18	0.014*	0.217108	0.588549	0.65664	0.237827	0.442985	0.512883	0.839446
bio19	0.021*	0.237373	0.631901	0.665708	0.280523	0.427636	0.642133	0.721112
PH	0.646361	0.512676	0.82056	0.996	0.857569	0.258462	0.825928	0.665743
SOC	0.478841	0.608	0.362667	0.254979	0.113965	0.563027	0.268377	0.113965
TN	0.302703	0.608	0.371597	0.31915	0.060098	0.448	0.099556	0.0896

TP	0.805696	0.289032	0.099556	0.086857	0.015448276*	0.082783	0.512676	0.014*
TK	0.258462	0.456453	0.014*	0.051692	0.021*	0.839446	0.7168	0.570453
AN	0.254979	0.894918	0.608	0.553143	0.051692	0.608	-0.0311111118*	0.082783
AP	0.085787	0.996	0.635122	0.572609	0.247718	0.701702	0.65664	0.880151
AK	0.205639	0.049411765*	0.754213	0.979113	0.948945	0.320264	0.765137	0.216889
Elevation	-0.014*	0.368667	0.237827	0.258462	0.0154482768*	0.948945	0.237827	0.608

Significance levels (*P≤0.05) were used in the analyses.
